# Supplementary material for: Complexome profiling of the Chlamydomonas psb28 mutant reveals TEF5 as an early PSII assembly factor
Source: Plant Cell. 2025 Mar 17;37(6):koaf055. doi: 10.1093/plcell/koaf055 (PMC12164590; doi:10.1093/plcell/koaf055)
Supplement: koaf055_Supplementary_Data [file koaf055_supplementary_data.zip › psb28_interactive.zip!/plots/intensity/qualityPlots/peptides/CPLD49_Cre16.g666050.t1.2.html]

### Cre16.g666050.t1.2

Trivial name: CPLD49  
SeqCoverage [median]: 10.00  
Mass: 48.62 kDa  
  
 

| Sequence | MissedCleavage | Mass [Da] | wtIntensity | muIntensity | Score |
| --- | --- | --- | --- | --- | --- |
| SVVVIGGTGR | 0 | 943.55 | 770.80 | 1995.04 | 76.68 |
| TPAPGESVEPK | 0 | 1110.56 | 434.59 | 0.00 | 54.42 |
| VDITNADSVQALLK | 0 | 1485.80 | 1119.98 | 1316.36 | 117.23 |
|  |  |  | 2325.4 | 3311.4 |  |
